# RNA splicing and splicing regulator changes in prostate cancer pathology

**DOI:** 10.1007/s00439-017-1792-9

**Published:** 2017-04-05

**Authors:** Jennifer Munkley, Karen Livermore, Prabhakar Rajan, David J. Elliott

**Affiliations:** 1 Institute of Genetic Medicine, Newcastle University, Central Parkway, Newcastle, NE1 3BZ England, UK; 2 Barts Cancer Institute, John Vane Science Centre, Queen Mary University of London, Charterhouse Square, London, EC1M 6BQ UK

## Abstract

Changes in mRNA splice patterns have been associated with key pathological mechanisms in prostate cancer progression. The androgen receptor (abbreviated AR) transcription factor is a major driver of prostate cancer pathology and activated by androgen steroid hormones. Selection of alternative promoters by the activated AR can critically alter gene function by switching mRNA isoform production, including creating a pro-oncogenic isoform of the normally tumour suppressor gene *TSC2*. A number of androgen-regulated genes generate alternatively spliced mRNA isoforms, including a prostate-specific splice isoform of *ST6GALNAC1* mRNA. *ST6GALNAC1* encodes a sialyltransferase that catalyses the synthesis of the cancer-associated sTn antigen important for cell mobility. Genetic rearrangements occurring early in prostate cancer development place *ERG* oncogene expression under the control of the androgen-regulated *TMPRSS2* promoter to hijack cell behaviour. This *TMPRSS2*–*ERG* fusion gene shows different patterns of alternative splicing in invasive versus localised prostate cancer. Alternative *AR* mRNA isoforms play a key role in the generation of prostate cancer drug resistance, by providing a mechanism through which prostate cancer cells can grow in limited serum androgen concentrations. A number of splicing regulator proteins change expression patterns in prostate cancer and may help drive key stages of disease progression. Up-regulation of SRRM4 establishes neuronal splicing patterns in neuroendocrine prostate cancer. The splicing regulators Sam68 and Tra2β increase expression in prostate cancer. The SR protein kinase SRPK1 that modulates the activity of SR proteins is up-regulated in prostate cancer and has already given encouraging results as a potential therapeutic target in mouse models.

## Introduction

Alternative mRNA isoforms have important roles in normal development and physiology. Almost every human gene produces more than one mRNA isoform, vastly expanding the information content of the human genome (Djebali et al. 2012). Alternative and aberrant pre-mRNA splice isoforms also play an important role in cancer. In fact, altered splicing patterns have been suggested as a new “hallmark” of cancer cells, in addition to other well-established hallmarks of cancer such as evasion of cell death and metastasis (Hanahan and Weinberg 2011; Ladomery 2013; Oltean and Bates 2014). Recent data indicate a key role for splicing pattern changes in the pathology of prostate cancer. Alternative splicing programmes in prostate cancer have been the topic of excellent reviews (Hagen and Ladomery 2012; Lu et al. 2015; Nakazawa et al. 2014; Rajan et al. 2009b; Sette 2013). Here we particularly concentrate on developments in the last 3 years.

**Fig. 1:**
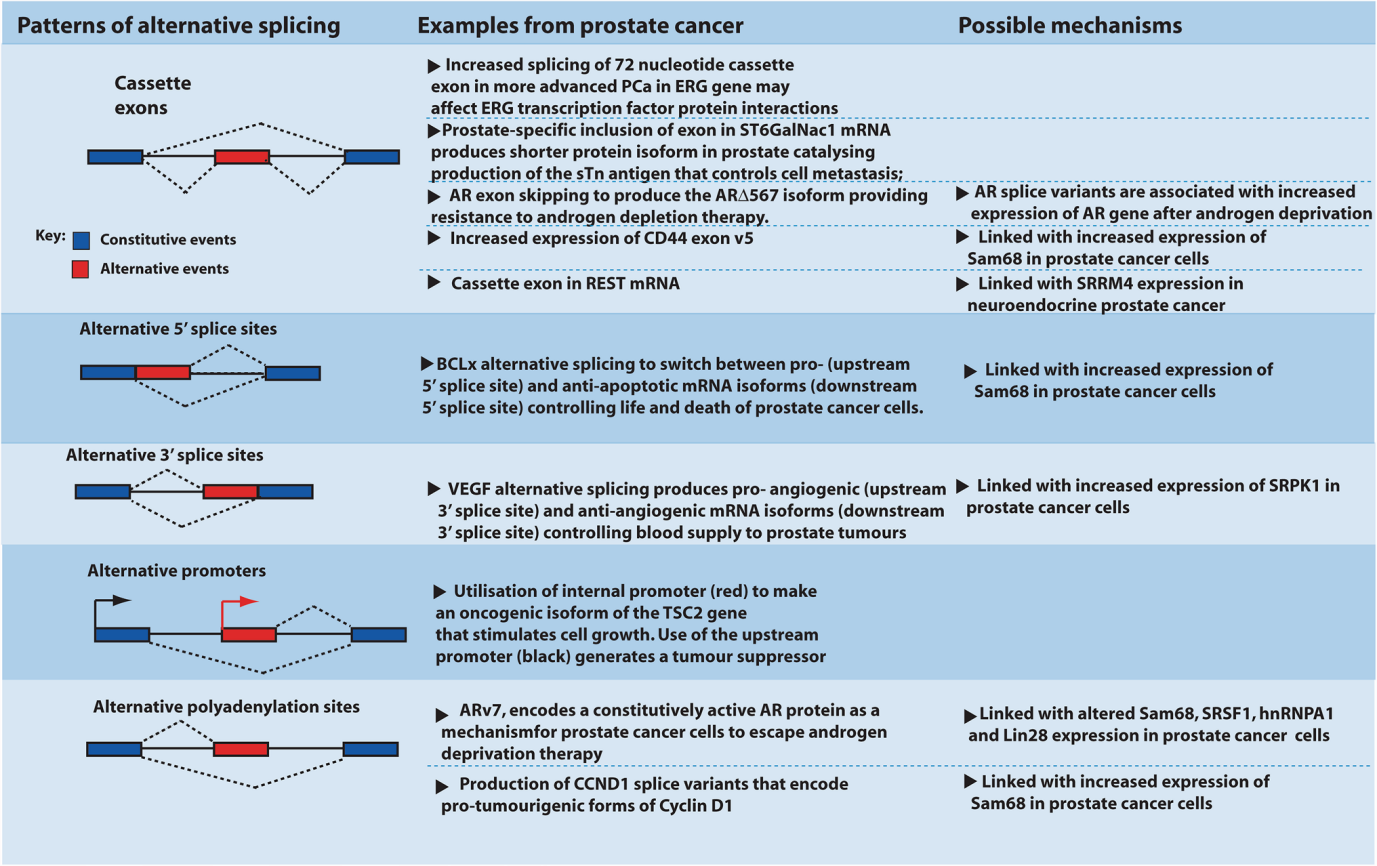
Different kinds of splicing pattern and their effect on prostate cancer cell biology. The most common form of alternative splicing in human cells is shown, with key examples from prostate cancer (not shown, whole introns can also be left in the mRNA)

Alternative splicing patterns include whole exons being either spliced in or left out (exon skipping) and alternative utilisation of both 5′ and 3′ splice sites to insert exons of different sizes. Alternative mRNA isoforms can also be generated by the selection of different promoters and different polyadenylation sites. Each of these pathways produces mRNA splice variants that can impact on prostate cancer development or response to treatment (summarised in Fig. 1).

**Fig. 2:**
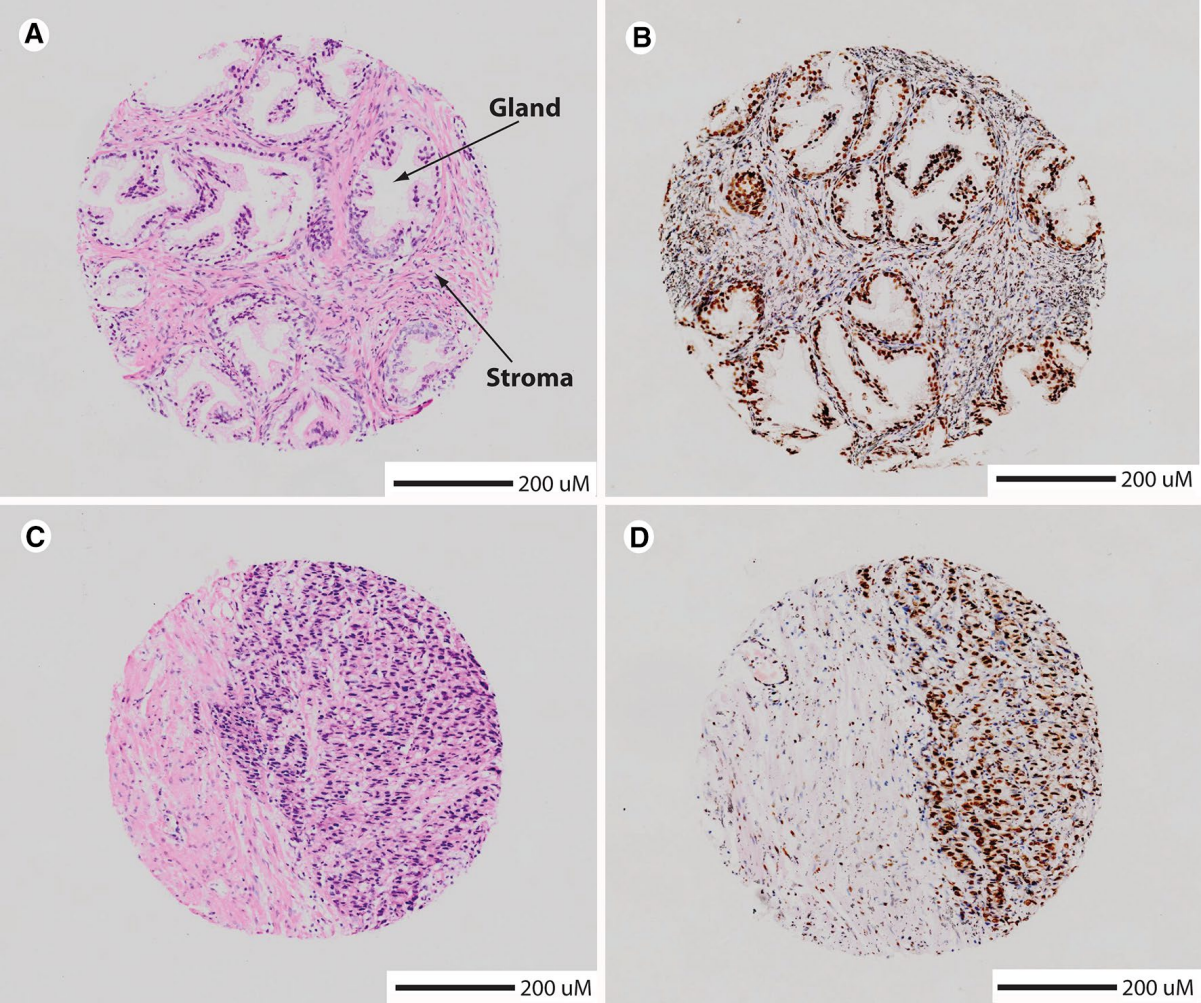
Prostate tissue visualised using tissue biopsies. **a**, **b**. Histological sections made from benign prostatic hyperplasia (BPH, with normal glandular structure embedded in stroma). Prostate cancer development is clinically described as a series of Gleason grades (1–5, with 1 corresponding to well-differentiated tissue containing a glandular structure and 5 being the most advanced with only few glands still visible) (Gleason and Mellinger 1974; Mellinger et al. 1967). **c**, **d** Histological sections made from prostate cancer (Gleason grade 5, notice breakdown of glandular structure). Left panels, sections processed using H&E staining. Right panels, sections processed by staining with haematoxylin and counterstaining with the RNA-binding protein Sam68 (*brown stain*). Figure adapted from (Rajan et al. 2008) with permission from the Journal of Pathology

Prostate cancer is the second most frequent male cancer in the UK, with 47,300 cases being diagnosed in the UK in 2014 (corresponding to 130 new cases diagnosed per day, http://www.cancerresearchuk.org/health-professional/cancer-statistics/statistics-by-cancer-type/prostate-cancer#heading-Zero). The prostate is a small gland located just below the bladder that produces seminal fluid components. Prostate cancers typically do not cause symptoms, but in some more advanced stages can block urine flow from the bladder, invade the adjacent seminal vesicles and metastasise more distantly to bone. Primary prostate cancer leads to a breakdown in the normal glandular structure of the prostate gland. Prostate cancer is classified histologically by morphological features using the Gleason scoring system (Gleason and Mellinger 1974) (for example, see Fig. 2).

## Alternative splicing of genes under androgen control in prostate cancer

**Fig. 3:**
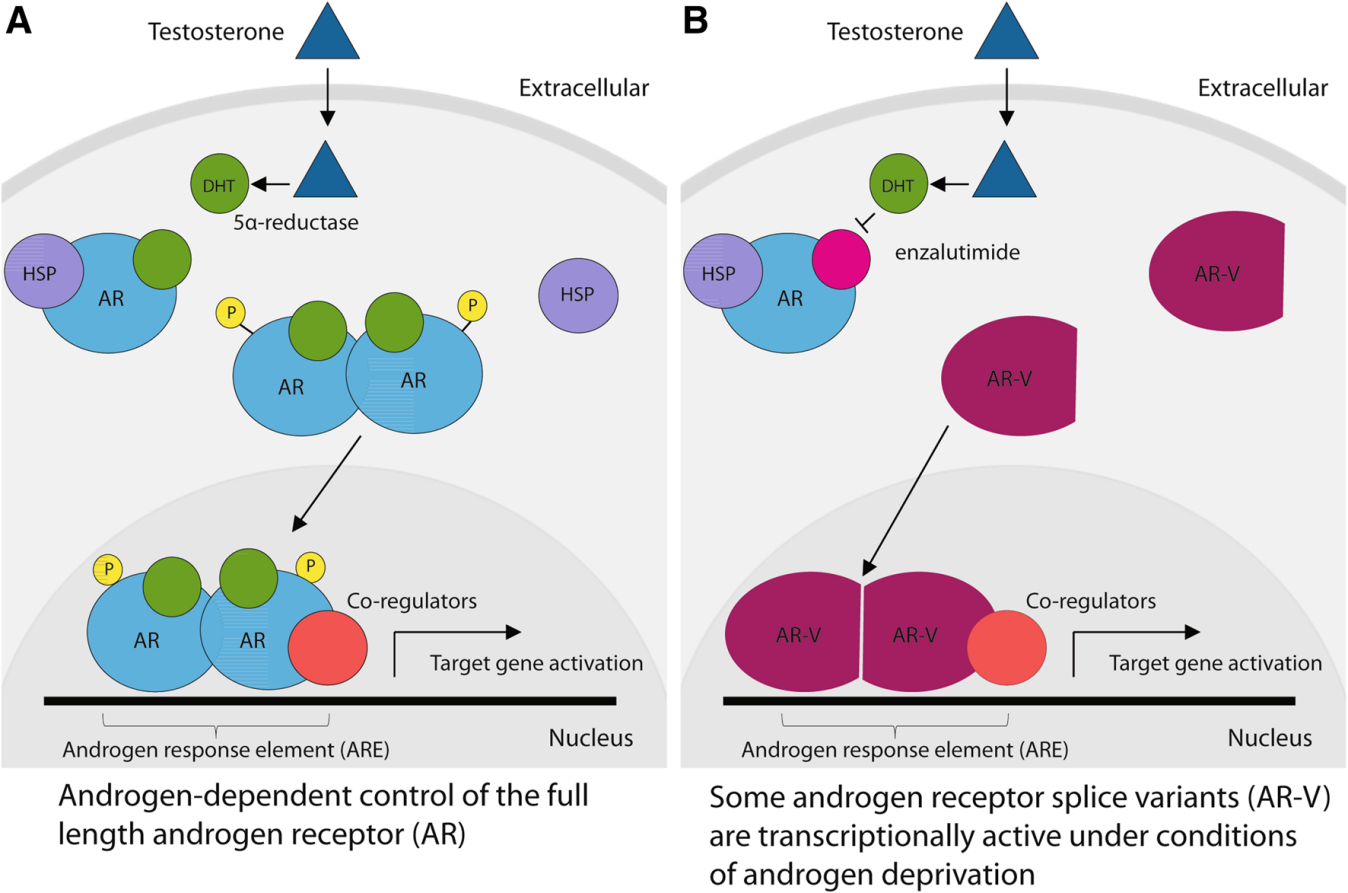
Transcriptional control by **a** the full-length androgen receptor and **b** constitutively active AR isoforms made by splice variants. In (**a**), testosterone enters the prostate cancer cell and becomes modified to dihydroxytestosterone (DHT) by 5α-reductase. DHT binds to the androgen receptor (AR), displacing heat shock protein 90 (HSP) and resulting in AR translocation into the nucleus. Once inside the nucleus, the AR binds to consensus DNA sequence elements called androgen response elements (AREs) to control target gene expression. In (**b**), an androgen receptor variant protein (AR-V) lacking the ligand-binding domain is able to directly translocate into the nucleus without binding to DHT, resulting in androgen-independent control of gene expression

Clinical progression of prostate cancer is fuelled by a group of steroid hormones called androgens (Livermore 2016; Mills 2014). Androgens are small hydrophobic molecules that can cross the cell membrane, and include the male hormone testosterone. Once within cells, androgens bind to a nuclear hormone receptor protein called the androgen receptor (AR). The AR typically has a default cytoplasmic location in the absence of androgens, but translocates into the nucleus after binding to androgens via its ligand-binding domain (LBD). Once inside the nucleus, the AR binds to DNA target sequences via its DNA-binding domain (DBD) and controls patterns of downstream gene transcription via its N-terminal TF domain (Fig. 3a). The AR transcriptionally controls in the order of 700 genes within prostate cancer cells (Munkley et al. 2016).

Androgen hormones can affect splicing patterns as well as transcription. Many splicing decisions are made on nascent RNAs while their transcription is still in progress (Kornblihtt 2006). Increased transcriptional speeds from androgen-regulated promoters in the presence of androgens could potentially provide the spliceosome with a choice of exons to include into the mRNA. Consistent with this, transcripts from a *CD44* minigene driven from a steroid-responsive promoter show increased exon skipping in response to the joint presence of the AR and androgens (Rajan et al. 2008). The AR also recruits some RNA-binding proteins as cofactors that can affect splicing. These include the RNA-binding protein Sam68 that decreases skipping of *CD44* variable exons, and the RNA helicase p68 that increases skipping of *CD44* variable exons (Fig. 1) (Clark et al. 2008; Rajan et al. 2008).

The above experiments used artificial model minigenes to investigate splicing control of AR target genes. Initial searches to find endogenous androgen-dependent splice isoforms used exon microarrays to probe the entire transcriptome (Rajan et al. 2011). These initial experiments identified two endogenous androgen-dependent cassette exons, one in the *ZNF121* gene that encodes a zinc finger-containing protein (this *ZNF121* exon was activated by androgens) and one in the *NDUFV3* gene that encodes a mitochondrial respiratory protein (this *NDUFV3* exon was repressed by androgens). Although these *ZNF121* and *NDUFV3* exons changed splicing in response to androgens, their clinical importance is not known, nor if these genes are direct targets for the AR. However, this same study also identified a number of androgen-dependent mRNAs made from alternative promoters, including an alternative mRNA isoform of the normally tumour suppressor *TSC2* (*Tuberous Sclerosis 2*) gene that is transcribed from an internal promoter (so only contains downstream *TSC2* exons). The well-characterised full-length TSC2 protein represses cell growth via the mTOR pathway. The androgen-driven alternative *TSC2* mRNA isoform encodes a shorter (C-terminal only) TSC2 protein that promotes rather than represses cell growth (Munkley et al. 2014).

More recent RNAseq analysis of prostate cancer cell transcriptomes identified an androgen-regulated and prostate-specific *ST6GALNAC1* mRNA splice isoform. *ST6GALNAC1* encodes the important ST6GalNac1 enzyme that synthesises the cancer-associated sialyl-Tn (sTn) antigen (Munkley et al. 2015). In prostate cancer cells, an alternative spliced *ST6GALNAC1* mRNA isoform that encodes a shorter ST6GalNac1 protein isoform is made. This shorter ST6GalNac1 protein isoform is actually made from a longer mRNA, since an extra exon is included within the 5′ untranslated region (UTR) of the *ST6GALNAC1* mRNA in the prostate. The presence of this extra exon causes an alternative start codon to be utilised, resulting in the shorter version of the ST6GalNac1 protein (Munkley et al. 2015). This shorter ST6GalNac1 protein is produced at higher levels in prostate cancer cells than the previously reported full-length protein; yet it is able to synthesise the sTn antigen which is linked to patient survival and metastasis, and controls cell adhesion (Munkley et al. 2015). The changed 5′ UTR structure of the prostate-specific mRNA isoform of ST6GalNac1 might even enhance its translation, resulting in increased ST6GalNAc1 enzyme levels and more synthesis of sTn antigen (Munkley 2016). Both the short and long isoforms of ST6GalNac1 use the same androgen-driven promoter, but the 5′ UTR exon-skipped isoform has only been reported thus far in the prostate.

Alternative splicing patterns of an androgen-regulated oncogenic fusion gene called *ETS*-*related gene* (*ERG*) are associated with more advanced forms of prostate cancer progression. *ERG* is a proto-oncogene that plays a key role in the pathology of prostate cancer. *ERG* encodes a transcription factor that controls the expression of many genes during normal development (Adamo and Ladomery 2016). *ERG* is not normally transcriptionally controlled by androgens, but gene fusions can place *ERG* under transcriptional control by the androgen-regulated *TMPRSS2* gene on the transition between prostatic epithelial neoplasia to prostate carcinoma. Such genetic rearrangements frequently link exon 1 or exons 1 and 2 of the *TMPRSS2* gene, and the upstream *TMPRSS2* gene promoter, to exon 4 and downstream regions of the *ERG* gene, by removing intervening regions of chromosome 21.

The *TMPRSS2*–*ERG* fusion gene is one of the most frequently over-expressed genes in prostate cancer. The encoded TMPRSS2–ERG fusion protein controls many important properties of prostate cancer cells, including cytoskeletal organisation, cell proliferation, expression of prostate-specific antigen (abbreviated PSA; this is a key serum biomarker for detecting prostate cancer) and epithelial–mesenchymal transitions (EMT) crucial for prostate cancer cell metastasis (Adamo and Ladomery 2016). Reverse transcription PCR (RT-PCR) analyses of tumour mRNA from patients show that more clinically advanced prostate cancers with histological evidence for seminal gland invasion have decreased skipping of two cassette exons within the *ERG* gene, compared with more localised prostate cancers and benign prostate tissue (Hagen et al. 2014). Increased splicing inclusion of these two *ERG* exons might contribute to the production of more oncogenic isoforms of the ERG fusion protein. One of these alternatively spliced *ERG* exons (of 72 nucleotides in length) encodes an in-frame peptide sequence. Inclusion of this 72-nucleotide exon might affect the interactions of the encoded ERG protein with transcription factors and other nuclear proteins, since it is immediately adjacent to the region of the *ETS* gene that encodes a protein–protein interaction domain called the sterile alpha motif (SAM)/pointed domain (http://pfam.xfam.org/family/SAM_PNT).

## Changes in *AR* mRNA splicing patterns enable prostate cancer cells to become hormone resistant

The primary therapeutic strategy for advanced prostate cancer treatment is to block androgen signalling through androgen deprivation therapy or AR blockade, thereby halting tumour progression. Abiraterone is a drug that inhibits androgen biosynthesis and so reduces the levels of androgens within prostate cancer cells. Enzalutamide is a drug that antagonises the interaction of androgens with AR protein. Although prostate tumours initially respond to androgen deprivation therapy, later stages of the disease can develop into a treatment-resistant form of the disease called castration-resistant prostate cancer. Mechanisms of prostate cancer cell resistance cross over, so a prostate cancer cell developing resistance to enzalutamide will also show a reduced (~20%) response to abiraterone, and vice versa (Liu et al. 2016).

Changing patterns of *AR* pre-mRNA splicing play a critical role in enabling prostate cancer cells to develop castration resistance. Changed splicing patterns generate variant AR protein isoforms (abbreviated AR-V) that lack ligand-binding domains, frequently as a result of splicing inclusion of cryptic exons (abbreviated CE), making them independent of androgen control. Some AR-V protein variants translocate into the nucleus even in the presence of enzalutamide (Fig. 3). *AR* pre-mRNA splicing changes thus enable prostate cancer cells to proliferate during androgen deprivation therapy in reduced circulating androgen concentrations.

**Fig. 4:**
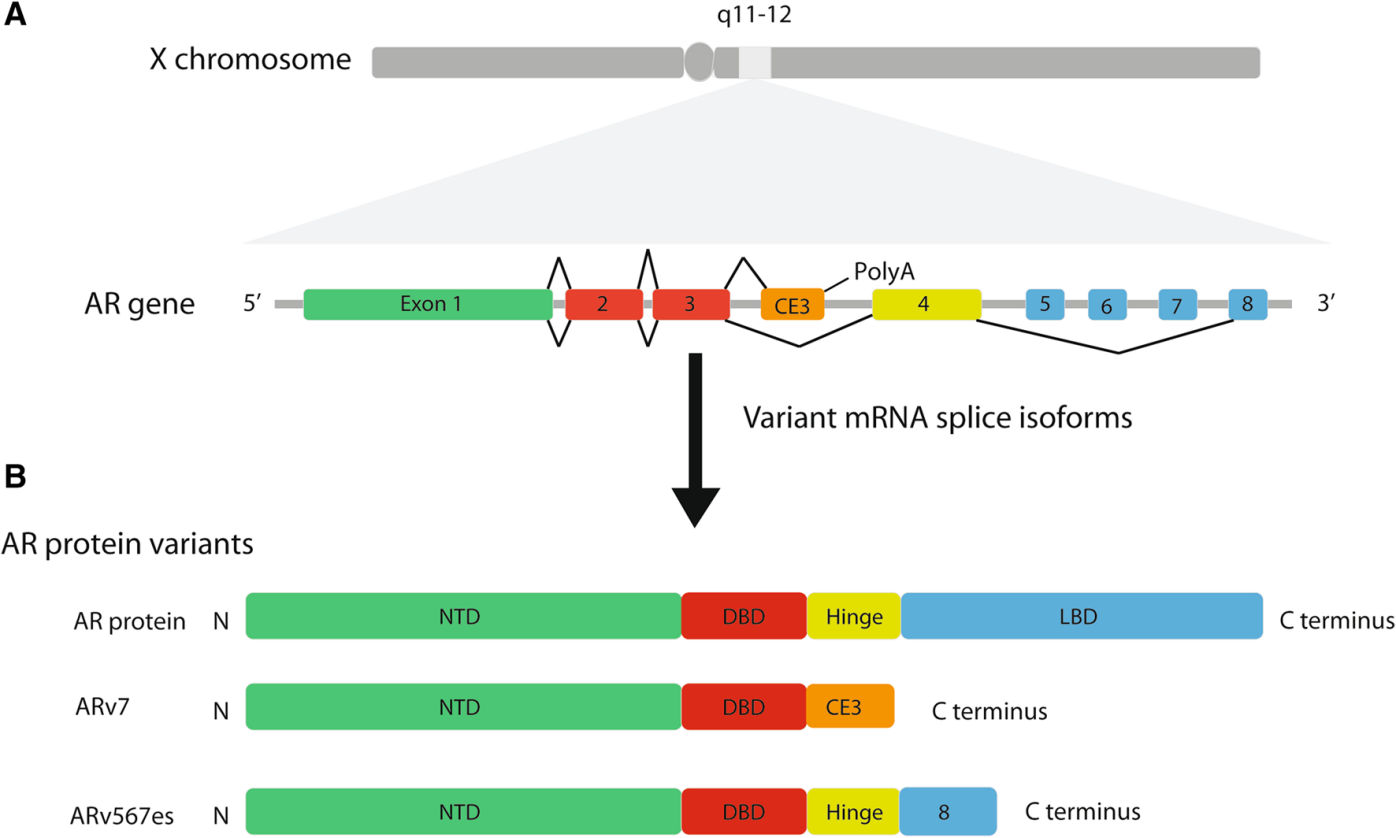
Exon–intron organisation of the *AR* gene and frequent pathogenic *AR* mRNA splice isoforms. **a** The AR protein is encoded by the 8-exon *AR* gene on the X chromosome. The full-length AR mRNA is made by splicing of exons 1–8. ARv7 mRNA is made by splicing cryptic exon 3 (CE3) after exons 1–3. Splicing of CE3 is associated with transcriptional termination, so this makes a truncated mRNA and truncated AR protein (aberrant splicing pathway shown above the AR gene). ARv567 is made by skipping of exons 5–7 in the *AR* mRNA (aberrant splicing pathway shown below the AR gene). Exons 1–8 are spliced together to produce the canonical *AR* splice isoform that encodes a full-length AR protein isoform. Aberrant splicing patterns include splicing of exon 3 to cryptic exon CE3, which is linked to premature termination of transcription (using an intron 3-internal polyA site) and a truncated mRNA, and skipping of exons 5–7. **b** Both full-length and AR variants are translated from different mRNA isoforms. Full-length AR protein contains an N-terminal domain (important for transcriptional activation) encoded by exon 1, a C4-type zinc finger DNA-binding domain encoded by exons 2–3 and a ligand-binding domain (which binds to androgens) encoded by exons 4–8. ARv7 is lacking the ligand-binding domain. ARv567es is lacking most of the ligand-binding domain

Around 20 *AR* variant splice isoforms have been implicated in the development of hormone refractory prostate cancer [reviewed by Lu and Luo (2013)]. The clinically most frequent *AR* splice variant, called *ARv7*, is produced by splicing inclusion of a cryptic exon called CE3 located within intron 3 (Fig. 4). *AR* CE3 is a terminal exon, meaning that splicing inclusion is linked to the selection of a new poly(A) site, thus creating a truncated *AR* mRNA that lacks coding information for the Ligand-Binding Domain (abbreviated LBD). As a result, *ARv7* encodes a short yet constitutively active isoform of the AR (active in the absence of androgens). While full-length AR protein is dependent on androgen binding via its LBD to translocate into the nucleus and control transcriptional activity, ARv7 protein is constitutively present in the nucleus even in the absence of androgens and so can provide AR activity in androgen-depleted prostate cancer cells (Cao et al. 2014). Expression of ARv7 is important for prostate cancer cell growth: siRNA-mediated depletion of ARv7 (using siRNAs complementary to CE3) inhibits the growth of the VCaP cell line in androgen-limiting conditions (Liu et al. 2014b).

Supporting ARv7 protein or RNA expression being a potentially useful biomarker for disease progression, AR variant splice isoform levels change during prostate cancer development. Expression levels of ARv7 mRNA in patients with prostate cancer predict their pharmacological response to enzalutamide and abiraterone (Antonarakis et al. 2014). Levels of nuclear Arv7 protein can also be monitored by immunohistochemistry using a monoclonal antibody and are predictive of overall survival (Welti et al. 2016). Prostate cancer cells metastasise to bone via circulating tumour cells which are released from the primary tumour. Single-cell RNAseq analysis of circulating tumour cells purified from the bloodstream identified *AR* splice variants in most (8 out of 11 sequenced) patients, but less frequently in primary tumours (Miyamoto et al. 2015). Consistent with *ARv7* expression being an adaptive response of cells to reduced androgen levels, *ARv7* splice isoform levels increase in response to androgen deprivation therapy and decrease on the reintroduction of androgens (Yu et al. 2014). Expression of *ARv7* is controlled by the transcription factors Myc (Myc also controls the expression of full-length *AR*) and NFκβ2, both of which increase expression in prostate cancer (Nadiminty et al. 2015).

Rather than providing a like-for-like replacement with the full-length androgen receptor, ARv7 protein instead preferentially regulates the expression of genes involved in active cell division and so promotes cell division rather than differentiation (Hu et al. 2012; Nakazawa et al. 2014). ARv7 expression in the LNCaP prostate cancer cell line also changes patterns of cell metabolism, decreasing the production of citrate and increasing the breakdown of glutamine—features of the “Warburg effect” changes in cancer metabolism that are also observed in prostate tumours (Shafi et al. 2015).

*ARv567es* is a further cancer-associated *AR* splice form observed in patients and also encodes a constitutively active AR protein (Fig. 4). *ARv567es* mRNA is made through skipping of exons 5–7 of the *AR* pre-mRNA and is only expressed within prostate cancer (and not the normal prostate). The ARv567es protein isoform regulates the transcription of genes involved in cell cycle control and particularly activates the expression of the oncogene *UBE2C*. UBE2C is a ubiquitin-conjugating protein active during mitosis, as part of the anaphase-promoting complex (APC) that inactivates the mitotic checkpoint control. UBE2C protein is highly expressed in solid tumours and promotes cell proliferation. Transcriptional activation by the ARv567es AR isoform occurs via a DNA looping mechanism that involves interaction with the transcription factor MED1 (part of the mediator complex involved in transcriptional initiation), and within castration-resistant but not hormone-responsive prostate cancer (Liu et al. 2015). In mice, the expression of AR variant isoforms can be sufficient themselves to induce cancer development. Transgenic mouse models expressing ARv567es and ARv7 proteins within their prostate glands develop prostate cancer (Liu et al. 2013; Sun et al. 2014).

## AR splice variants and the pathways that generate them are therapeutic targets in prostate cancer

The clinical importance of aberrant *AR* splice isoforms has generated a lot of interest in their generation. A good model for investigating this is the 22Rv1 prostate cancer cell line, which expresses ARv7 protein as well as full-length AR protein. Depletion of ARv7 is sufficient to sensitise 22Rv1 cells to abiraterone (Liu et al. 2016). A genomic deletion removes 48 nucleotides of intron 1 from the *AR* gene within 22Rv1 cells. This genomic deletion might contribute to altered splicing patterns of CE3, through changing intronic binding sites available for splicing regulator proteins (Li et al. 2012).

Androgen deprivation therapy results in increased binding of SRSF1 and U2AF65 near the 3′ splice site of cryptic CE3, although the levels of these splicing regulator proteins themselves do not change in response to androgen deprivation (Liu et al. 2014b). However, the expression levels of *U2AF2* (the gene which encodes U2AF65) do increase in hormone-resistant prostate cancer compared to primary prostate cancer (Stockley et al. 2015). Expression levels of the splicing regulator Sam68 protein also increase in prostate cancer (Busa et al. 2007; Rajan et al. 2008) and can drive *AR* CE3 splicing inclusion (Stockley et al. 2015). ARv7 splicing production is also stimulated by the expression of the splicing regulator hnRNPA1 (Nadiminty et al. 2015). This is particularly important, since the levels of hnRNPA1 increase in prostate cancer, and hnRNPA1 expression has been observed as an early marker for tumour development in several cancers. Genomic rearrangements including intron sequences also occur in situ within prostate cancers expressing *AR* mRNA splice variants, although the major factor leading to the expression of AR splice variants is the corresponding increased expression levels of the full-length *AR* isoform (Henzler et al. 2016).

It is hoped that understanding the splicing pathways generating *AR* splice variants will lead to downstream clinical applications used to treat prostate cancer. Supporting this idea, siRNA-mediated depletion of hnRNA1 re-sensitises a cell line model of castration-resistant prostate cancer to enzalutamide (Nadiminty et al. 2015). Thus, the connection between hnRNPA1 expression and splicing pathways producing *ARv7* might prove to be clinically relevant. Interestingly, increased levels of the RNA-binding protein Lin28 also correlate with increased *ARv7* expression. Down-regulation of Lin28 sensitises prostate cancer cells to enzalutamide. Lin28 expression increases in prostate cancer and regulates the expression of *Let7* family microRNAs that operate upstream of Myc and a number of splicing factors including hnRNPA1 that in turn control *ARv7* production (Tummala et al. 2013, 2016).

Small-molecule inhibitors might be used to block splicing pathways leading to *ARv7* production. One such small molecule is Onalespib, which inhibits the heat shock protein HSP90. Ordinarily, translocation of full-length AR protein into the nucleus following androgen stimulation is achieved via interactions with HSP90 (Fig. 3a). Onalespib does not affect the splicing pathway generating the full-length AR protein isoform (although full-length AR protein stability is decreased by Onalespib). Onalespib does inhibit the splicing pathways that generate the ARv7 splice isoform (in addition to >500 other splice isoforms) (Ferraldeschi et al. 2016). In an in vivo model, Onalespib treatment also increases survival of nude mice xenografted with the ARv7-expressing 22Rv1 cell line. Mechanistically, HSP90 inhibition in prostate cancer cells might affect signalling pathways operating upstream of SR proteins like SRSF1 that are involved in *AR* CE3 splicing inclusion (possibly including effects on the protein kinase SRPK1, see below). However, it is worth noting that other HSP90 inhibitors have been reported to have different effects on *ARv7* splice isoform variant production (Gillis et al. 2013; Shafi et al. 2015).

Alternative splicing patterns could help guide the development of personalised therapies for prostate cancer patients (Anand and Bjartell 2015; Antonarakis et al. 2014; Palapattu 2016; Savage 2015; Sun and Abdollah 2015; Taneja 2015). Since the production of ARv7 is predictive of abiraterone and enzalutamide sensitivity in advanced prostate cancer, this may guide treatment options towards selection of other drug regimes that should be used instead. Such drugs include taxanes (which inhibit microtubule function) and galeterone (targets the AR for degradation, as well as blocking binding of androgens) (Antonarakis et al. 2014, 2015; Onstenk et al. 2015; Savage 2015; Thadani-Mulero et al. 2014). An FDA-approved drug called niclosamide, originally developed against helminths, strongly targets ARv7 and restores susceptibility of prostate cancer cells to abiraterone, both in vitro and by oral administration into nude mice with 22Rv1 cell line xenografts (Liu et al. 2014a, 2016).

## Splicing regulators can change expression patterns in prostate cancer

A number of splicing regulator proteins change expression patterns in prostate cancer, including in neuroendocrine prostate cancer. Neuroendocrine prostate cancer is an aggressive form of prostate cancer thought to result from trans-differentiation of castration-resistant prostate cancer cells into cells harbouring a neuroendocrine phenotype. This transition occurs at low androgen conditions. Neuroendocrine prostate cancer does not respond to AR-directed therapies. Recent data indicate that the development of neuroendocrine prostate cancer is driven by an increased expression of the splicing factor SRRM4 (serine/arginine repetitive matrix 4 protein). SRRM4 is a splicing factor normally needed in the body (outside of the prostate gland) for neural differentiation. SRRM4 up-regulation drives splicing switches towards neuronal splicing patterns and the trans-differentiation of prostate cells towards neuroendocrine prostate cancer cells (Li et al. 2016; Zhang et al. 2015). Important SRRM4-regulated events include a splicing switch in the transcription factor REST1 (RE1 silencing transcription factor) which is a master regulator of neurogenesis, thus providing a possible molecular explanation for the neuroendocrine prostate cancer phenotype (Li et al. 2016).

The KH domain-containing protein Sam68 is also up-regulated in prostate cancer (Busa et al. 2007; Rajan et al. 2008). Sam68 interacts with RNA via its STAR domain [comprising a KH domain and flanking protein sequences (Feracci et al. 2016)]. Sam68 regulates splicing of the *CD44* mRNA (controlling the production of splice isoforms important in metastasis), *BCLx* (controlling splice isoforms important in cell death via apoptosis) and *CCND1* (controlling splice isoforms important in the cell cycle) [Fig. 1, and reviewed by (Sette 2013)]. Recent work shows that Sam68 protein interacts with the transcriptional co-activator protein SND1, to switch splicing of *CD44* pre-mRNA towards the production of more metastasis-associated splice isoforms [including *CD44* exon v5 (Cappellari et al. 2014)]. Levels of both SND1 and Sam68 protein increase in prostate cancer cells. Sam68 also interacts with the transcription factor FBI-1 to control *BCLx* splicing patterns, with FBI-1 inhibiting the production of the apoptotic BCLx isoform (Bielli et al. 2014). Sam68 protein additionally interacts with the RNA splicing regulator hnRNPA2/B1, which is also up-regulated in prostate cancer, and controls the expression of β-catenin (Rajan et al. 2009a; Stockley et al. 2014).

The splicing regulator protein Tra2β also increases the expression in prostate cancer tissues, and this is associated with preoperative prostate-specific antigen, lymph node metastasis, clinical stage, Gleason score and biochemical recurrence (Diao et al. 2015). Global studies carried out in breast cancer cells discovered *CHK1* exon 3 as an important splicing target for Tra2β (Best et al. 2014). CHK1 is a key checkpoint protein that monitors DNA integrity after replication stress. Interestingly, joint depletion of Tra2β and its ortholog Tra2α inhibits *CHK1* exon 3 splicing in the LNCaP prostate cancer cell line (Best et al. 2014). Since *CHK1* exon 3 is not a multiple of 3, this skipping event leads to loss of function, accumulation of DNA damage and cell death.

Another splicing regulator that changes expression in prostate cancer is the protein kinase SRPK1. SRPK1 does not itself bind to pre-mRNA directly, but instead phosphorylates members of the important SR family of splicing regulators, including SRSF1. SRSF1 is a critical splicing regulator in the cell, and phosphorylation by SRPK1 enables SRSF1 nuclear localisation. Once in the nucleus, SRSF1 controls splicing of *VEGF* (vascular epithelial growth factor) mRNA which is important for angiogenesis. SRSF1 switches *VEGF* splicing patterns between pro- and anti-angiogenic mRNA splice isoforms via selection of an alternative 3′ splice site (Fig. 1) (Amin et al. 2011). SRPK1 is a promising target for therapy. SRPK1 inhibition keeps SRSF1 in the cytoplasm and, as a result, decreases the ability of the prostate cancer cell line PC-3 to form tumours in mouse xenografts, probably through changed patterns of *VEGF* mRNA splicing depriving prostate cancer cells from developing a blood supply (Mavrou et al. 2015). SRPK1 expression increases in prostate cancer (Bullock and Oltean 2016; Bullock et al. 2016; Mavrou et al. 2015). Existing drugs are available that can target SRPK1 and have already been tested in mouse models (Mavrou et al. 2015; Mavrou and Oltean 2016). A further important kinase that might impact on prostate cancer pathology is aurora A, which affects the production of *ARv* splice variants, likely also through control of SRSF1 (Jones et al. 2017).

## Current developments

Investigation of splicing in prostate cancer is currently undergoing methodological change with the incorporation of analysis from big data sets, particularly with the global discovery of new splice isoforms. Future research in this area is likely to include comparison of large “-omics” level datasets to detect many splice isoform changes between different grades of prostate cancer tissue in parallel. Already, RNAseq datasets from patients with prostate cancer are available on sites like The Cancer Genome Atlas (TCGA, https://portal.gdc.cancer.gov/) and the International Cancer Genome Consortia (ICGC, http://icgc.org/). Increased understanding of mRNA splice isoforms in prostate cancer will also come from integrating RNAseq data with peptide data from mass spectroscopy, thus enabling direct correlation of alternative isoforms and their encoded proteins (Evans et al. 2012; Nesvizhskii 2014). Individual splice isoform functions can be dissected to assess their importance in disease pathology, and whether they might be useful targets for therapeutic intervention or as prognostic markers (in a similar way in which the AR splice variants have been investigated).

Integrating data from system-wide approaches will also be helpful for understanding how these splice isoforms are generated in prostate cancer cells. Transcriptome-wide binding maps of RNA splicing regulator proteins in cancer cells can be used to identify splicing targets important for therapy and diagnostics (Best et al. 2014; Sundararaman et al. 2016). The expression patterns of these RNA splicing regulators themselves can also be globally analysed through transcriptome data, and at the genome level how these expression levels are impacted by copy number variation patterns of their genes (Sebestyen et al. 2016). Since splicing takes place co-transcriptionally on nascent pre-mRNAs, other contributors to alternative splicing patterns to be investigated in prostate cancer cells will be chromatin components and epigenetic marks (Allemand et al. 2016). These features might be particularly important also to functions of the AR in both prostate cancer pathology and generation of alternative splice isoforms (Rajan et al. 2009b, 2011).

## Summary

Although research in this area has been very productive, still remaining open in this field is a full understanding of how alternative splicing programmes impact on prostate cancer pathology. Research in the past few years shows that splicing patterns change during prostate cancer progression and in response to therapeutic intervention. Some splicing changes enable prostate cancer cells to grow in limiting concentrations of androgens, and some cause the production of more oncogenic proteins contributing to disease pathology. Understanding these splicing changes, and the mechanisms driving them, opens up new possibilities for developing diagnostic and therapeutic strategies to treat prostate cancer. As a result of these investigations, future therapies might aim to target specific splice isoforms, for example using antisense oligonucleotide and morpholino techniques that have been developed for other diseases including Duchenne muscular dystrophy and spinal muscular atrophy (Aartsma-Rus and Krieg 2017; Rigo et al. 2012). For example, blocking splicing of exon 3 of *CHK1* mRNA might specifically kill cancer cells that rely on *CHK1* in the absence of other checkpoints to control replication stress (Best et al. 2014, 2016).

## References

[CR1] Aartsma-Rus A, Krieg AM (2017). FDA approves eteplirsen for Duchenne muscular dystrophy: the next chapter in the Eteplirsen Saga. Nucleic Acid Ther.

[CR2] Adamo P, Ladomery MR (2016). The oncogene ERG: a key factor in prostate cancer. Oncogene.

[CR3] Allemand E, Myers MP, Garcia-Bernardo J, Harel-Bellan A, Krainer AR, Muchardt C (2016). A broad set of chromatin factors influences splicing. PLoS Genet.

[CR4] Amin EM, Oltean S, Hua J, Gammons MV, Hamdollah-Zadeh M, Welsh GI, Cheung MK, Ni L, Kase S, Rennel ES, Symonds KE, Nowak DG, Royer-Pokora B, Saleem MA, Hagiwara M, Schumacher VA, Harper SJ, Hinton DR, Bates DO, Ladomery MR (2011). WT1 mutants reveal SRPK1 to be a downstream angiogenesis target by altering VEGF splicing. Cancer Cell.

[CR5] Anand AU, Bjartell A (2015). Re: AR-V7 and resistance to enzalutamide and abiraterone in prostate cancer. Eur Urol.

[CR6] Antonarakis ES, Lu C, Wang H, Luber B, Nakazawa M, Roeser JC, Chen Y, Mohammad TA, Fedor HL, Lotan TL, Zheng Q, De Marzo AM, Isaacs JT, Isaacs WB, Nadal R, Paller CJ, Denmeade SR, Carducci MA, Eisenberger MA, Luo J (2014). AR-V7 and resistance to enzalutamide and abiraterone in prostate cancer. N Engl J Med.

[CR7] Antonarakis ES, Lu C, Luber B, Wang H, Chen Y, Nakazawa M, Nadal R, Paller CJ, Denmeade SR, Carducci MA, Eisenberger MA, Luo J (2015). Androgen receptor splice variant 7 and efficacy of taxane chemotherapy in patients with metastatic castration-resistant prostate cancer. JAMA Oncol.

[CR8] Best A, James K, Dalgliesh C, Hong E, Kheirolahi-Kouhestani M, Curk T, Xu Y, Danilenko M, Hussain R, Keavney B, Wipat A, Klinck R, Cowell IG, Cheong Lee K, Austin CA, Venables JP, Chabot B, Santibanez Koref M, Tyson-Capper A, Elliott DJ (2014). Human Tra2 proteins jointly control a CHEK1 splicing switch among alternative and constitutive target exons. Nat Commun.

[CR9] Best A, James K, Hysenaj G, Tyson-Capper A, Elliott DJ (2016). Transformer2 proteins protect breast cancer cells from accumulating replication stress by ensuring productive splicing of checkpoint kinase 1. Front Chem Sci Eng.

[CR10] Bielli P, Busa R, Di Stasi SM, Munoz MJ, Botti F, Kornblihtt AR, Sette C (2014). The transcription factor FBI-1 inhibits SAM68-mediated BCL-X alternative splicing and apoptosis. EMBO Rep.

[CR11] Bullock N, Oltean S (2016). The many faces of SRPK1. J Pathol.

[CR12] Bullock N, Potts J, Simpkin AJ, Koupparis A, Harper SJ, Oxley J, Oltean S (2016). Serine-arginine protein kinase 1 (SRPK1), a determinant of angiogenesis, is upregulated in prostate cancer and correlates with disease stage and invasion. J Clin Pathol.

[CR13] Busa R, Paronetto MP, Farini D, Pierantozzi E, Botti F, Angelini DF, Attisani F, Vespasiani G, Sette C (2007). The RNA-binding protein Sam68 contributes to proliferation and survival of human prostate cancer cells. Oncogene.

[CR14] Cao B, Qi Y, Zhang G, Xu D, Zhan Y, Alvarez X, Guo Z, Fu X, Plymate SR, Sartor O, Zhang H, Dong Y (2014). Androgen receptor splice variants activating the full-length receptor in mediating resistance to androgen-directed therapy. Oncotarget.

[CR15] Cappellari M, Bielli P, Paronetto MP, Ciccosanti F, Fimia GM, Saarikettu J, Silvennoinen O, Sette C (2014). The transcriptional co-activator SND1 is a novel regulator of alternative splicing in prostate cancer cells. Oncogene.

[CR16] Clark EL, Coulson A, Dalgliesh C, Rajan P, Nicol SM, Fleming S, Heer R, Gaughan L, Leung HY, Elliott DJ, Fuller-Pace FV, Robson CN (2008). The RNA helicase p68 is a novel androgen receptor coactivator involved in splicing and is overexpressed in prostate cancer. Cancer Res.

[CR17] Diao Y, Wu D, Dai Z, Kang H, Wang Z, Wang X (2015). Prognostic value of transformer 2beta expression in prostate cancer. Int J Clin Exp Pathol.

[CR18] Djebali S, Davis CA, Merkel A, Dobin A, Lassmann T, Mortazavi A, Tanzer A, Lagarde J, Lin W, Schlesinger F, Xue C, Marinov GK, Khatun J, Williams BA, Zaleski C, Rozowsky J, Roder M, Kokocinski F, Abdelhamid RF, Alioto T, Antoshechkin I, Baer MT, Bar NS, Batut P, Bell K, Bell I, Chakrabortty S, Chen X, Chrast J, Curado J, Derrien T, Drenkow J, Dumais E, Dumais J, Duttagupta R, Falconnet E, Fastuca M, Fejes-Toth K, Ferreira P, Foissac S, Fullwood MJ, Gao H, Gonzalez D, Gordon A, Gunawardena H, Howald C, Jha S, Johnson R, Kapranov P, King B, Kingswood C, Luo OJ, Park E, Persaud K, Preall JB, Ribeca P, Risk B, Robyr D, Sammeth M, Schaffer L, See LH, Shahab A, Skancke J, Suzuki AM, Takahashi H, Tilgner H, Trout D, Walters N, Wang H, Wrobel J, Yu Y, Ruan X, Hayashizaki Y, Harrow J, Gerstein M, Hubbard T, Reymond A, Antonarakis SE, Hannon G, Giddings MC, Ruan Y, Wold B, Carninci P, Guigo R, Gingeras TR (2012). Landscape of transcription in human cells. Nature.

[CR19] Evans VC, Barker G, Heesom KJ, Fan J, Bessant C, Matthews DA (2012). De novo derivation of proteomes from transcriptomes for transcript and protein identification. Nat Methods.

[CR20] Feracci M, Foot JN, Grellscheid SN, Danilenko M, Stehle R, Gonchar O, Kang HS, Dalgliesh C, Meyer NH, Liu Y, Lahat A, Sattler M, Eperon IC, Elliott DJ, Dominguez C (2016). Structural basis of RNA recognition and dimerization by the STAR proteins T-STAR and Sam68. Nat Commun.

[CR21] Ferraldeschi R, Welti J, Powers MV, Yuan W, Smyth T, Seed G, Riisnaes R, Hedayat S, Wang H, Crespo M, Nava Rodrigues D, Figueiredo I, Miranda S, Carreira S, Lyons JF, Sharp S, Plymate SR, Attard G, Wallis N, Workman P, de Bono JS (2016). Second-generation HSP90 inhibitor Onalespib blocks mRNA splicing of androgen receptor variant 7 in prostate cancer cells. Cancer Res.

[CR22] Gillis JL, Selth LA, Centenera MM, Townley SL, Sun S, Plymate SR, Tilley WD, Butler LM (2013). Constitutively-active androgen receptor variants function independently of the HSP90 chaperone but do not confer resistance to HSP90 inhibitors. Oncotarget.

[CR23] Gleason DF, Mellinger GT (1974). Prediction of prognosis for prostatic adenocarcinoma by combined histological grading and clinical staging. J Urol.

[CR24] Hagen RM, Ladomery MR (2012). Role of splice variants in the metastatic progression of prostate cancer. Biochem Soc Trans.

[CR25] Hagen RM, Adamo P, Karamat S, Oxley J, Aning JJ, Gillatt D, Persad R, Ladomery MR, Rhodes A (2014). Quantitative analysis of ERG expression and its splice isoforms in formalin-fixed, paraffin-embedded prostate cancer samples: association with seminal vesicle invasion and biochemical recurrence. Am J Clin Pathol.

[CR26] Hanahan D, Weinberg RA (2011). Hallmarks of cancer: the next generation. Cell.

[CR27] Henzler C, Li Y, Yang R, McBride T, Ho Y, Sprenger C, Liu G, Coleman I, Lakely B, Li R, Ma S, Landman SR, Kumar V, Hwang TH, Raj GV, Higano CS, Morrissey C, Nelson PS, Plymate SR, Dehm SM (2016). Truncation and constitutive activation of the androgen receptor by diverse genomic rearrangements in prostate cancer. Nat Commun.

[CR28] Hu R, Lu C, Mostaghel EA, Yegnasubramanian S, Gurel M, Tannahill C, Edwards J, Isaacs WB, Nelson PS, Bluemn E, Plymate SR, Luo J (2012). Distinct transcriptional programs mediated by the ligand-dependent full-length androgen receptor and its splice variants in castration-resistant prostate cancer. Cancer Res.

[CR29] Jones D, Noble M, Wedge SR, Robson CN, Gaughan L (2017). Aurora A regulates expression of AR-V7 in models of castrate resistant prostate cancer. Sci Rep.

[CR30] Kornblihtt AR (2006). Chromatin, transcript elongation and alternative splicing. Nat Struct Mol Biol.

[CR31] Ladomery M (2013). Aberrant alternative splicing is another hallmark of cancer. Int J Cell Biol.

[CR32] Li Y, Hwang TH, Oseth LA, Hauge A, Vessella RL, Schmechel SC, Hirsch B, Beckman KB, Silverstein KA, Dehm SM (2012). AR intragenic deletions linked to androgen receptor splice variant expression and activity in models of prostate cancer progression. Oncogene.

[CR33] Li Y, Donmez N, Sahinalp C, Xie N, Wang Y, Xue H, Mo F, Beltran H, Gleave M, Collins C, Dong X (2016). SRRM4 drives neuroendocrine transdifferentiation of prostate adenocarcinoma under androgen receptor pathway inhibition. Eur Urol.

[CR34] Liu G, Sprenger C, Sun S, Epilepsia KS, Haugk K, Zhang X, Coleman I, Nelson PS, Plymate S (2013). AR variant ARv567es induces carcinogenesis in a novel transgenic mouse model of prostate cancer. Neoplasia.

[CR35] Liu C, Lou W, Zhu Y, Nadiminty N, Schwartz CT, Evans CP, Gao AC (2014). Niclosamide inhibits androgen receptor variants expression and overcomes enzalutamide resistance in castration-resistant prostate cancer. Clin Cancer Res.

[CR36] Liu LL, Xie N, Sun S, Plymate S, Mostaghel E, Dong X (2014). Mechanisms of the androgen receptor splicing in prostate cancer cells. Oncogene.

[CR37] Liu G, Sprenger C, Wu PJ, Sun S, Uo T, Haugk K, Epilepsia KS, Plymate S (2015). MED1 mediates androgen receptor splice variant induced gene expression in the absence of ligand. Oncotarget.

[CR38] Liu C, Armstrong C, Zhu Y, Lou W, Gao AC (2016). Niclosamide enhances abiraterone treatment via inhibition of androgen receptor variants in castration resistant prostate cancer. Oncotarget.

[CR39] Livermore KE (2016). Androgen receptor and prostate cancer. AIMS Mol Sci.

[CR40] Lu C, Luo J (2013). Decoding the androgen receptor splice variants. Transl Androl Urol.

[CR41] Lu J, Van der Steen T, Tindall DJ (2015). Are androgen receptor variants a substitute for the full-length receptor?. Nat Rev Urol.

[CR42] Mavrou A, Oltean S (2016). SRPK1 inhibition in prostate cancer: a novel anti-angiogenic treatment through modulation of VEGF alternative splicing. Pharmacol Res.

[CR43] Mavrou A, Brakspear K, Hamdollah-Zadeh M, Damodaran G, Babaei-Jadidi R, Oxley J, Gillatt DA, Ladomery MR, Harper SJ, Bates DO, Oltean S (2015). Serine-arginine protein kinase 1 (SRPK1) inhibition as a potential novel targeted therapeutic strategy in prostate cancer. Oncogene.

[CR44] Mellinger GT, Gleason D, Bailar J (1967). The histology and prognosis of prostatic cancer. J Urol.

[CR45] Mills IG (2014). Maintaining and reprogramming genomic androgen receptor activity in prostate cancer. Nat Rev Cancer.

[CR46] Miyamoto DT, Zheng Y, Wittner BS, Lee RJ, Zhu H, Broderick KT, Desai R, Fox DB, Brannigan BW, Trautwein J, Arora KS, Desai N, Dahl DM, Sequist LV, Smith MR, Kapur R, Wu CL, Shioda T, Ramaswamy S, Ting DT, Toner M, Maheswaran S, Haber DA (2015). RNA-Seq of single prostate CTCs implicates noncanonical Wnt signaling in antiandrogen resistance. Science.

[CR47] Munkley J (2016). The role of sialyl-Tn in cancer. Int J Mol Sci.

[CR48] Munkley J, Rajan P, Lafferty NP, Dalgliesh C, Jackson RM, Robson CN, Leung HY, Elliott DJ (2014). A novel androgen-regulated isoform of the TSC2 tumour suppressor gene increases cell proliferation. Oncotarget.

[CR49] Munkley J, Oltean S, Vodak D, Wilson BT, Livermore KE, Zhou Y, Star E, Floros VI, Johannessen B, Knight B, McCullagh P, McGrath J, Crundwell M, Skotheim RI, Robson CN, Leung HY, Harries LW, Rajan P, Mills IG, Elliott DJ (2015). The androgen receptor controls expression of the cancer-associated sTn antigen and cell adhesion through induction of ST6GalNAc1 in prostate cancer. Oncotarget.

[CR50] Munkley J, Vodak D, Livermore KE, James K, Wilson BT, Knight B, McCullagh P, McGrath J, Crundwell M, Harries LW, Leung HY, Robson CN, Mills IG, Rajan P, Elliott DJ (2016). Glycosylation is an androgen-regulated process essential for prostate cancer cell viability. EBioMedicine.

[CR51] Nadiminty N, Tummala R, Liu C, Lou W, Evans CP, Gao AC (2015). NF-kappaB2/p52:c-Myc:hnRNPA1 pathway regulates expression of androgen receptor splice variants and enzalutamide sensitivity in prostate cancer. Mol Cancer Ther.

[CR52] Nakazawa M, Antonarakis ES, Luo J (2014). Androgen receptor splice variants in the era of enzalutamide and abiraterone. Horm Cancer.

[CR53] Nesvizhskii AI (2014). Proteogenomics: concepts, applications and computational strategies. Nat Methods.

[CR54] Oltean S, Bates DO (2014). Hallmarks of alternative splicing in cancer. Oncogene.

[CR55] Onstenk W, Sieuwerts AM, Kraan J, Van M, Nieuweboer AJ, Mathijssen RH, Hamberg P, Meulenbeld HJ, De Laere B, Dirix LY, van Soest RJ, Lolkema MP, Martens JW, van Weerden WM, Jenster GW, Foekens JA, de Wit R, Sleijfer S (2015). Efficacy of cabazitaxel in castration-resistant prostate cancer is independent of the presence of AR-V7 in circulating tumor cells. Eur Urol.

[CR56] Palapattu GS (2016). Commentary on “AR-V7 and resistance to enzalutamide and abiraterone in prostate cancer.” Antonarakis ES, Lu C, Wang H, Luber B, Nakazawa M, Roeser JC, Chen Y, Mohammad TA, Chen Y, Fedor HL, Lotan TL, Zheng Q, De Marzo AM, Isaacs JT, Isaacs WB, Nadal R, Paller CJ, Denmeade SR, Carducci MA, Eisenberger MA, Luo J, Division of Urologic Oncology, Department of Urology, University of Michigan, MI. N Engl J Med 2014; 371(11):1028–38. Urol Oncol..

[CR57] Rajan P, Gaughan L, Dalgliesh C, El-Sherif A, Robson CN, Leung HY, Elliott DJ (2008). The RNA-binding and adaptor protein Sam68 modulates signal-dependent splicing and transcriptional activity of the androgen receptor. J Pathol.

[CR58] Rajan P, Dalgliesh C, Bourgeois CF, Heiner M, Emami K, Clark EL, Bindereif A, Stevenin J, Robson CN, Leung HY, Elliott DJ (2009). Proteomic identification of heterogeneous nuclear ribonucleoprotein L as a novel component of SLM/Sam68 nuclear bodies. BMC Cell Biol.

[CR59] Rajan P, Elliott DJ, Robson CN, Leung HY (2009). Alternative splicing and biological heterogeneity in prostate cancer. Nat Rev Urol.

[CR60] Rajan P, Dalgliesh C, Carling PJ, Buist T, Zhang C, Grellscheid SN, Armstrong K, Stockley J, Simillion C, Gaughan L, Kalna G, Zhang MQ, Robson CN, Leung HY, Elliott DJ (2011). Identification of novel androgen-regulated pathways and mRNA isoforms through genome-wide exon-specific profiling of the LNCaP transcriptome. PLoS ONE.

[CR61] Rigo F, Hua Y, Krainer AR, Bennett CF (2012). Antisense-based therapy for the treatment of spinal muscular atrophy. J Cell Biol.

[CR62] Savage N (2015). Metastasis: resistance fighters. Nature.

[CR63] Sebestyen E, Singh B, Minana B, Pages A, Mateo F, Pujana MA, Valcarcel J, Eyras E (2016). Large-scale analysis of genome and transcriptome alterations in multiple tumors unveils novel cancer-relevant splicing networks. Genome Res.

[CR64] Sette C (2013). Alternative splicing programs in prostate cancer. Int J Cell Biol.

[CR65] Shafi AA, Putluri V, Arnold JM, Tsouko E, Maity S, Roberts JM, Coarfa C, Frigo DE, Putluri N, Sreekumar A, Weigel NL (2015). Differential regulation of metabolic pathways by androgen receptor (AR) and its constitutively active splice variant, AR-V7, in prostate cancer cells. Oncotarget.

[CR66] Stockley J, Villasevil ME, Nixon C, Ahmad I, Leung HY, Rajan P (2014). The RNA-binding protein hnRNPA2 regulates beta-catenin protein expression and is overexpressed in prostate cancer. RNA Biol.

[CR67] Stockley J, Markert E, Zhou Y, Robson CN, Elliott DJ, Lindberg J, Leung HY, Rajan P (2015). The RNA-binding protein Sam68 regulates expression and transcription function of the androgen receptor splice variant AR-V7. Sci Rep.

[CR68] Sun M, Abdollah F (2015). Re: AR-V7 and Resistance to Enzalutamide and Abiraterone in Prostate Cancer. Eur Urol.

[CR69] Sun F, Chen HG, Li W, Yang X, Wang X, Jiang R, Guo Z, Chen H, Huang J, Borowsky AD, Qiu Y (2014). Androgen receptor splice variant AR3 promotes prostate cancer via modulating expression of autocrine/paracrine factors. J Biol Chem.

[CR70] Sundararaman B, Zhan L, Blue SM, Stanton R, Elkins K, Olson S, Wei X, Van Nostrand EL, Pratt GA, Huelga SC, Smalec BM, Wang X, Hong EL, Davidson JM, Lecuyer E, Graveley BR, Yeo GW (2016). Resources for the comprehensive discovery of functional RNA elements. Mol Cell.

[CR71] Taneja SS (2015). Re: AR-V7 and resistance to enzalutamide and abiraterone in prostate cancer. J Urol.

[CR72] Thadani-Mulero M, Portella L, Sun S, Sung M, Matov A, Vessella RL, Corey E, Nanus DM, Plymate SR, Giannakakou P (2014). Androgen receptor splice variants determine taxane sensitivity in prostate cancer. Cancer Res.

[CR73] Tummala R, Nadiminty N, Lou W, Zhu Y, Gandour-Edwards R, Chen HW, Evans CP, Gao AC (2013). Lin28 promotes growth of prostate cancer cells and activates the androgen receptor. Am J Pathol.

[CR74] Tummala R, Nadiminty N, Lou W, Evans CP, Gao AC (2016). Lin28 induces resistance to anti-androgens via promotion of AR splice variant generation. Prostate.

[CR75] Welti J, Rodrigues DN, Sharp A, Sun S, Lorente D, Riisnaes R, Figueiredo I, Zafeiriou Z, Rescigno P, de Bono JS, Plymate SR (2016). Analytical validation and clinical qualification of a new immunohistochemical assay for androgen receptor splice variant-7 protein expression in metastatic castration-resistant prostate cancer. Eur Urol.

[CR76] Yu Z, Chen S, Sowalsky AG, Voznesensky OS, Mostaghel EA, Nelson PS, Cai C, Balk SP (2014). Rapid induction of androgen receptor splice variants by androgen deprivation in prostate cancer. Clin Cancer Res.

[CR77] Zhang X, Coleman IM, Brown LG, True LD, Kollath L, Lucas JM, Lam HM, Dumpit R, Corey E, Chery L, Lakely B, Higano CS, Montgomery B, Roudier M, Lange PH, Nelson PS, Vessella RL, Morrissey C (2015). SRRM4 expression and the loss of REST activity may promote the emergence of the neuroendocrine phenotype in castration-resistant prostate cancer. Clin Cancer Res.

